# Functional outcome and prognostic factors in anti-Jo1 patients with antisynthetase syndrome

**DOI:** 10.1186/ar4332

**Published:** 2013-10-08

**Authors:** Isabelle Marie, Pierre-Yves Hatron, Patrick Cherin, Eric Hachulla, Elisabeth Diot, Olivier Vittecoq, Jean-François Menard, Fabienne Jouen, Stéphane Dominique

**Affiliations:** 1 Department of Internal Medicine, CHU Rouen, 76031, Rouen Cedex, France; 2 INSERM U 905, University of Rouen IFRMP, Institute for Biochemical Research, 76031, Rouen Cedex, France; 3 Department of Internal Medicine, CHU Lille, 59000, Lille Cedex, France; 4 Department of Internal Medicine, Pitié-Salpêtrière Hospital, 75651, Paris Cedex, France; 5 Department of Internal Medicine, CHU Tours, 37000, Tours Cedex, France; 6 Department of Rheumatology, CHU Rouen, 76031, Rouen Cedex, France; 7 Department of Biostatistics, CHU Rouen, 76031, Rouen Cedex, France; 8 Laboratory of Immunology, CHU Rouen and INSERM U 905 University of Rouen IFRMP, Institute for Biochemical Research, 76031, Rouen Cedex, France; 9 Department of Pneumology, CHU Rouen, 76031, Rouen Cedex, France

## Abstract

**Introduction:**

The aims of this present study were firstly to assess the outcome, including functional course, in anti-Jo1 positive patients with antisynthetase syndrome (ASS), and secondly to determine predictive parameters of poor outcome in these patients.

**Methods:**

The medical records of 86 consecutive anti-Jo1 patients with ASS were reviewed in 4 academic centers.

**Results:**

13 patients (15.1%) achieved remission of ASS, whereas 55 (63.9%) improved and 18 (20.9%) deteriorated in their clinical status. Both steroid and cytotoxic drugs could be discontinued in only 4.7% of patients. ASS was associated with decreased quality of life at long-term follow-up: only 69.2% of patients considered to be in remission experienced a return to previous normal activities; and 24.7% of other patients with non-remitting ASS still had a marked reduction of activities (as shown by the disability scale of the Health Assessment Questionnaire). Decreased quality of life was further due to calcinosis cutis (8.1%) and adverse effects of steroid therapy (36%). Factors associated with ASS deterioration were older age, pulmonary and esophageal involvement, calcinosis cutis and cancer. Higher anti-Jo1 levels were further associated with disease severity in ASS patients.

**Conclusions:**

The present study shows high morbidity related to ASS. Furthermore, we suggest that patients with predictive factors of ASS deterioration may require more aggressive therapy. Our findings also suggest that in anti-Jo1 patients with severe esophageal manifestations, combined high dose steroids and intravenous immunoglobulins might be proposed as the first line therapy. Finally, as cancer occurred in 14% of anti-Jo1 patients, our findings underscore that the search for cancer should be performed in these patients.

## Introduction

Antisynthetase syndrome (ASS) is characterized by polymyositis/dermatomyositis (PM/DM) associated with antisynthetase antibodies, fever, arthritis, Raynaud’s phenomenon, mechanic’s hands and interstitial lung disease (ILD) [1-3]. The anti-Jo1 antibody is the most common of the antisynthetase antibodies (60% to 80%) [1-4].

ASS is still considered to be associated with high morbidity rates, principally related to muscle weakness and lung complications [4-7]. Nevertheless, to date, only a few series have analyzed the outcome and prognostic factors in anti-Jo1 patients with ASS. The aims of the current study were to (1) assess outcomes, including functional course, in 86 anti-Jo1 patients with ASS; and (2) determine predictive parameters of poor outcomes in these patients.

## Methods

Our retrospective study began with a search of the participating institutional centers’ medical record indexes, which provided us access to the diagnoses of the centers’ patients. The first electronic search involved use of the PM/DM codes to identify patients with a diagnosis of PM/DM who were seen as either inpatients or outpatients between 1996 and 2010 at four academic centers (Lille, Paris, Rouen and Tours). The diagnosis of PM/DM was based on Bohan and Peter criteria [8,9], and only patients with definite or probable disease were included. During the study period, 346 consecutive patients were seen for evaluation of PM/DM. All patients had been tested for anti-Jo1 antibody using immunodiffusion with subsequent confirmation by enzyme-linked immunosorbent assay. A second search was used to isolate the subset of anti-Jo1-positive patients with ASS. Eighty-six anti-Jo1 patients with ASS were identified. None of these patients had other connective tissue disorders or myopathy. The data from all patients were anonymously reported. This retrospective study was approved by the institutional ethics committee of CPP de Haute-Normandie with a waiver for informed consent.

### Initial evaluation of patients

At the time of diagnosis, muscle weakness, involving both upper and lower limbs and neck extensors and flexors, was assessed by manual muscle strength testing (MMT) using the UK Medical Research Council Scale (0 to 5), with a theoretical maximum score of 85 points (that is, normal muscle power).

All patients had an initial evaluation for organ involvement, which resulted in the detection of the following systemic complications:

1. Raynaud’s phenomenon.

2. Mechanic’s hands.

3. Joint impairment: arthralgia, arthritis (painful and/or swollen joints) and deforming arthropathy characterized by subluxation of interphalangeal joints of the thumbs and periarticular hydroxyapatite calcifications.

4. Esophageal dysfunction: The diagnosis of PM/DM-related esophageal involvement was based on the following:

4a. The presence of clinical manifestations, that is, dysphagia, gastroesophageal reflux into the pharynx and/or the mouth, coughing while eating, and aphagia for solids and liquids.

4a. Findings of esophageal manometry: Esophageal motor impairment related to PM/DM was diagnosed by the presence of at least one of the following findings: low pressure in the upper esophageal sphincter, decreased or absent peristalsis in the upper third of the esophageal body and decreased peristalsis within the lower two-thirds of the esophageal body.

4a. Gastroscopy, which was performed to exclude esophageal mucosal involvement that might be responsible for dysphagia that proved normal.

5. Respiratory muscle involvement resulting from ventilatory insufficiency related to striated muscle weakness, when patients had ventilatory failure with decreased vital capacity: Ventilatory insufficiency due to respiratory striated muscle weakness was dichotomized as previously described [10]. Severe hypoventilation was determined by hypercapnic respiratory failure requiring mechanical ventilation, and moderate hypoventilation was characterized by a restrictive pattern on pulmonary function tests (PFTs) (decreased lung volumes with vital capacity less than 80%).

6. Aspiration pneumonia.

7. Pulmonary arterial hypertension diagnosed on the basis of echocardiography.

In addition, patients were examined for underlying malignancy.

ILD involvement was systematically investigated initially by PFTs and high-resolution computed tomography (HRCT) scans of the lungs. The diagnosis of ILD was made if patients had (1) PFT abnormalities manifested by restrictive changes (vital capacity less than 80%) and diffusing capacity of carbon monoxide less than 70% of the predicted value [11] and (2) radiographic abnormalities consistent with ILD on HRCT scans of the lungs [3,12]. Because HRCT patterns have been correlated with pulmonary histological findings, patients with ILD were divided into three groups based on HRCT predominant patterns indicative of cryptogenic organizing pneumonia (COP) characterized by consolidation and linear opacities, nonspecific interstitial pneumonia (NSIP) characterized by ground-glass opacities and irregular linear opacities, and usual interstitial pneumonia (UIP) characterized by honeycombing and traction bronchiectases [3,12].

### Patient outcomes

Patients had a minimal follow-up of 18 months, although patients who died before the 18-month follow-up were also included. The functional course of all patients was assessed for muscle functional disability according to the disability scale of the Health Assessment Questionnaire (HAQ), which is divided into eight variables: dressing and grooming, arising, eating, walking, hygiene, reach, grip and activity [13].

The outcome of myositis was defined as (1) remission, characterized by stable increase or normalization of muscle strength (determined on the basis of MMT), skin changes and serum muscle enzyme levels (creatine kinase, CK), all persisting after therapy discontinuation; (2) improvement, with therapy, in muscle signs (on the basis of MMT) and skin changes and decreased CK levels; and (3) deterioration, despite therapy, when muscle signs (on the basis of MMT) and skin changes worsened and CK levels increased [10]. ASS was defined as (1) monocyclic when patients remained free of all clinical and biochemical signs of disease activity at 24-month follow-up after ASS diagnosis and (2) chronic continuous when patients had a chronic, progressive course of ASS and continuation of medication beyond the 24-month follow-up after ASS diagnosis [10]. Recurrence of myositis was diagnosed on the basis of clinical relapse. Recurrence was divided into two types: (1) short-term, occurring during tapering of therapy; and (2) long-term, occurring after therapy termination [10].

In addition, the HAQ score [13] was used to evaluate functional disability of ASS patients at the last follow-up. Particular attention was further paid to the development of the following steroid-related complications: muscle weakness, osteoporotic vertebral fracture and avascular necrosis. These latter outcomes were evaluated on the basis of the clinical records available.

Both survival and current status were based on hospital records (that is, data obtained during routine follow-up visits). The causes of death were also derived from hospital and physician records.

### Measurement of prognostic factors

Prognostic factors were determined at the time of ASS diagnosis. We assessed factors associated with poor prognosis in ASS patients. Patients were divided into two groups: patients whose condition deteriorated due to ASS and those whose condition did not. For group comparisons involving binary data, we used either the χ^2^ test or Fisher’s exact test, depending on the expected cell count (more or less than 5, respectively). Comparisons involving continuous data were performed using the Mann–Whitney *U* test. The results were regarded as significant when the *P*-value was less than 0.05.

## Results

### General background

The 86 anti-Jo1 patients comprised 32 men and 54 women with a median age of 54 years (range: 17 to 79 years) at the time of ASS diagnosis. Fifty-one patients had PM, and thirty-five had DM. At the time of initial diagnosis of ASS, 74 patients presented with myalgia (86%) and 66 exhibited muscle weakness (76.7%). The median score for muscle power on the MMT was 66 (range: 48 to 83). The median HAQ score for the cohort at the time of ASS diagnosis was 0.9. HAQ-specific frequency scores in patients were as follows: 0 < HAQ ≤ 0.5 (*n* = 29), 0.5 < HAQ ≤ 1 (*n* = 27), 1 < HAQ ≤ 1.5 (*n* = 12), 1.5 < HAQ ≤ 2 (*n* = 9), 2 < HAQ ≤ 2.5 (*n* = 6) and 2.5 < HAQ ≤ 2.75 (*n* = 3).

The general clinical characteristics of anti-Jo1 patients are shown in Table 1. ASS patients exhibited Raynaud’s phenomenon (*n* = 42); mechanic’s hands (*n* = 26); joint impairment (*n* = 59), including deforming arthropathy of the hands (*n* = 3); esophageal dysfunction (*n* = 21) (Table 2); and calcinosis cutis (*n* = 7). They also exhibited the following pulmonary complications: (1) ILD (*n* = 55) (Table 2), two of whom developed pulmonary arterial hypertension related to ILD; (2) ventilatory insufficiency related to striated muscle weakness (*n* = 13), 12 of whom had moderate hypoventilation and 1 of whom exhibited severe hypoventilation requiring mechanical ventilation; and (3) aspiration pneumonia (*n* = 17) related to esophageal motor impairment (*n* = 12) and ventilatory insufficiency due to respiratory striated muscle weakness (*n* = 5).

**Table 1:** General characteristics of anti-Jo1 patients with antisynthetase syndrome

**Characteristics**	**Data (*N*** **= 86 patients)**
Median age, years (range)	54 (15 to 83)
Men/women (*n*)	32/54
Clinical manifestations, *n* (%)	
Raynaud’s phenomenon	42 (11%)
Mechanic’s hands	26 (30.2%)
Arthralgia/arthritis	59 (68.6%)
Esophageal involvement	21 (24.4%)
Pulmonary involvement	
Interstitial lung disease	55 (64%)
Ventilatory insufficiency	13 (15.1%)
Aspiration pneumonia	17 (19.8%)
Calcinosis cutis	7 (8.1%)
Malignancy	12 (13.9%)

**Table 2:** **Esophageal complications and interstitial lung disease characteristics of anti-Jo1-positive antisynthetase syndrome patients**^**a**^

**Complications and characteristics**	**Percentage ( *n *)**
Esophageal complications (*N* = 21)	
Time of onset	
Concomitant with PM/DM	19% (*n* = 4)
1 to 3 months after PM/DM diagnosis	28.6% (*n* = 6)
More than 3 months after PM/DM diagnosis	52.4% (*n* = 11)
Presenting symptoms	
Odynophagia	4.8% (*n* = 1)
Dysphagia	81% (*n* = 17)
GER into pharynx and/or mouth	33.3% (*n* = 7)
Coughing while eating	61.9% (*n* = 13)
Aphagia for solids and liquids	14.3% (*n* = 3)
Esophageal manometry	
Low pressure in upper esophageal sphincter	85.7% (*n* = 18)
Decreased peristalsis in upper third of esophageal body	85.7% (*n* = 18)
Absent peristalsis in upper third of esophageal body	14.3% (*n* = 3)
Decreased peristalsis in lower two-thirds of esophageal body	9.5% (*n* = 2)
Interstitial lung disease (*N* = 55)	
Time of onset	
Before PM/DM	10.9% (*n* = 6)
Concomitant with PM/DM	70.9% (*n* = 39)
After PM/DM	18.2% (*n* = 10)
PFT	
FVC (median)	74%
VC (median)	75%
DLCO (median)	61%
HRCT scan pattern	
COP	12.7% (*n* = 7)
NSIP	63.7% (*n* = 35)
UIP	23.6% (*n* = 13)

^a^COP: cryptogenic organizing pneumonia; DLCO: diffusing capacity of carbon monoxide; FVC: forced vital capacity; GER: gastroesophageal reflux; HRCT: high-resolution computed tomography; ILD: interstitial lung disease; NSIP: nonspecific interstitial pneumonia; PFT: pulmonary function test; PM/DM: polymyositis/dermatomyositis; UIP: usual interstitial pneumonia; VC vital capacity.

Twelve patients (seven with DM and five with PM) had cancer of the colon (*n* = 3), breast (*n* = 3), ovaries (*n* = 2), uterus (*n* = 1), lung (*n* = 2) or pancreas (*n* = 1). The median age of these patients at the time of cancer diagnosis was 61.5 years (range: 49 to 75 years). Cancer onset in these patients preceded myositis within three years before ASS diagnosis (*n* = 3), was concurrently identified in association with ASS (*n* = 7) or (3) developed within two years after ASS diagnosis (*n* = 2).

### Therapy

All 86 patients were given high-dose steroid therapy initially (1 mg/kg/day). Steroid therapy was started within three months after onset of ASS symptoms in 82.6% of the cases and within one year in 97.7%. Twenty-two patients (25.6%) received steroids as monotherapy. Other anti-Jo1 patients with steroid-refractory ASS were further treated with second-line immunosuppressive agents (74.4% of cases).

Thirty patients were treated with methotrexate (MTX), which resulted in improvement of ASS in 83.3% of cases. Twenty-five patients were treated with azathioprine (AZA), which resulted in improvement of ASS in 72% of cases. Thirty-nine patients were given intravenous immunoglobulins (IVIGs) (1 g/kg for two days/month; median duration: six months), which led to clinical improvement in 69.2% of cases. Twenty patients with esophageal involvement received IVIG therapy (1 g/kg/day for two days/month). The median IVIG therapy duration was six months (range: four to nine months). Because oral feeding was impossible in the three patients with aphagia for solids and liquids (Table 2), these patients also required percutaneous endoscopic gastrostomy (*n* = 1) or gastric intubation (*n* = 2). Eighteen (90%) of twenty IVIG-treated patients had reduction of esophageal manifestations. In these patients, a significant improvement in esophageal manifestations occurred within two weeks after the first IVIG regimen, and complete disappearance of esophageal impairment was observed within fifteen days after the second IVIG regimen. This efficacy resulted in return to normal oral feeding and ablation of oral feeding tubes in all cases. Seven patients with MTX/AZA-refractory ASS were given mycophenolate mofetil (MMF), which resulted in resolution (*n* = 1) or improvement (*n* = 3) of clinical signs (57.1% of cases). Nineteen patients were given cyclophosphamide (six cycles intravenously) for ASS-related ILD, leading to improvement and/or stabilization of lung status in 89.4% of cases. One patient with refractory myositis and ILD was successfully treated with rituximab as follows: 1 g at days 0 and 14 and 1 g every 6 months for 18 months.

### Outcomes

The median follow-up duration of patients was 45 months (range: 1 to 228 months). The outcomes of these patients are summarized in Table 3.

**Table 3:** **Outcomes of anti-Jo1 patients with antisynthetase syndrome**^**a**^

**Outcomes**	**Data, *n *** **(%)**
Resolution	13 (15.1%)
Improvement	55 (63.9%)
Deterioration	18 (20.9%)
ASS course	
Monocyclic	1 (1.2%)
Chronic continuous	56 (65.1%)
Recurrences	
During tapering of high-dose steroids	5 (5.8%)
During tapering of low-dose steroids	69 (80.2%)
Off treatment	1 (1.2%)
Mortality	10 (11.6%)

^a^ASS: antisynthetase syndrome.

Thirteen patients (15.1%) had clinical remission of ASS, and only one of these patients had monocyclic PM/DM. At the last follow-up, only four of these thirteen patients were receiving no therapy and the other nine patients were being treated with low-dose prednisone alone (*n* = 4), MTX (*n* = 3) or AZA (*n* = 2).

Fifty-five other patients (63.9%) had improvement of ASS. Forty-four of these patients had chronic continuous ASS. At the last follow-up, the median daily dose of prednisone was 10 mg in these patients. These patients concurrently received MTX (*n* = 20), AZA (*n* = 16), MMF (*n* = 5) and IVIG (*n* = 1); prednisone therapy could be disrupted for the other two patients, who still received cytotoxic drugs (MTX (*n* = 1) and MMF (*n* = 1)).

Eighteen patients (20.9%) had deterioration of ASS, and twelve had chronic continuous ASS. At the last follow-up, the median daily dose of prednisone was 17 mg in these patients. They also concurrently received AZA (*n* = 7), MTX (*n* = 4), MMF (*n* = 2) and IVIG (*n* = 3).

ASS recurred in 75 patients (87.2%). Short-term recurrence was encountered in 74 patients; of these patients, 69 received low-dose steroids (<20 mg/day). Long-term clinical recurrence of ASS occurred in one patient seven months after therapy termination.

Ten anti-Jo1 patients died, with nine ASS-specific deaths due to cancer (*n* = 5) or to pulmonary complications due to respiratory insufficiency related to ILD (*n* = 1) or aspiration pneumonia (*n* = 3). The longest time until death was 163 months after onset of ASS (median: 34 months (range: 0.1 to 163 months)).

### Long-term functional outcomes

The long-term functional outcomes of the patients who achieved ASS remission were characterized by complete disappearance of clinical manifestations with a return to previous normal activities in nine patients (69.2%), persistent muscle effort fatigue with moderate decreased activities (HAQ score < 0.75) in four patients and death due to disorders other than ASS complications in one patient. The long-term functional outcomes of the 73 remaining patients were persistent muscle effort fatigue with moderate decreased activities (HAQ score < 0.75) in 55 patients, marked decrease of muscle weakness with severe reduction of activities (0.75 < HAQ score < 1.5) in 15 patients and three patients who required a wheelchair (HAQ > 1.5).

The seven patients with calcinosis cutis developed the disabling complications of pain (*n* = 5), mechanical disturbances (*n* = 4), cosmetic disfigurement (*n* = 3) and ulceration with infection (*n* = 3). Calcinosis cutis was both extensive and diffuse in one of these patients. Patients also exhibited the steroid-related musculoskeletal complications of muscle weakness (*n* = 23) and osteoporotic vertebral fracture (*n* = 8).

### Factors associated with antisynthetase syndrome deterioration

We compared the characteristics of patients with or without ASS deterioration (Table 4). The median duration of patient follow-up did not differ between these two groups of patients (37 vs. 35 months; *P* = 0.673).

**Table 4:** **Clinical parameters associated with antisynthetase syndrome deterioration**^**a**^

**Characteristics**	**ASS remission/improvement**	**ASS deterioration**	***P***
	**(*n*** **= 68)**	**(*n*** **= 18)**	
General characteristics			
Median age, years (range)	60.5 (17 to 86)	53 (19 to 83)	0.002
Male/female ratio	39.7%/60.3%	27.8%/72.2%	0.420
PM/DM subset	61.8% PM/38.2% DM	50% PM/50% DM	0.424
Median duration of ASS symptoms before diagnosis, months (range)	3 (1 to 9)	4 (1 to 13)	0.680
Clinical characteristics			
Myalgia	82.4%	88.9%	1
Muscle weakness	72%	94.4%	0.05
Fever	16.2%	27.8%	0.309
Raynaud’s phenomenon	48.5%	50%	1
Mechanic’s hands	29.4%	33.3%	0.777
Joint manifestations	66.2%	77.8%	0.406
Esophageal involvement	19.1%	44.4%	0.03
Interstitial lung disease	61.8%	72.2%	0.582
Ventilatory insufficiency	10.3%	33.3%	0.02
Pyogenic pneumonia	14.7%	38.9%	0.04
Calcinosis cutis	2.9%	27.8%	0.004
Malignancy	7.4%	38.9%	0.002

^a^ASS: antisynthetase syndrome; PM/DM: polymyositis/dermatomyositis. *P* values were calculated using a χ^2^ test or Fisher’s exact test.

Patients who experienced ASS deterioration were older (60.5 years vs. 53 years; *P* = 0.002). We found no statistically significant difference between anti-Jo1 patients with or without ASS deterioration regarding sex (*P* = 0.420), PM/DM subset (*P* = 0.424), duration of clinical manifestations before therapy initiation (*P* = 0.884), myalgia (*P* = 1), Raynaud’s phenomenon (*P* = 1), mechanic’s hands (*P* = 0.767), joint involvement (*P* = 0.406) or ILD (*P* = 0.582).

Patients with ASS deterioration more commonly exhibited muscle weakness (*P* = 0.05) with a lower median score of muscle power on MMTs at initial diagnosis of ASS (62 vs. 71; *P* = 0.06), calcinosis cutis (*P* = 0.004), esophageal impairment (*P* = 0.03), ventilatory insufficiency related to striated muscle weakness (*P* = 0.02) and aspiration pneumonia (*P* = 0.04). We further found that patients with ASS deterioration, compared with those without it, presented lower median values of forced vital capacity (69.5% vs. 75.5%; *P* = 0.001) and vital capacity (68.5% vs. 75%; *P* = 0.001) at the time of ASS diagnosis. The UIP predominant pattern on HRCT scans was more frequent in the group of ILD patients with ASS deterioration (44.4% vs. 7.4%; *P* =0.0005). In addition, cancer was more frequent in the group of patients with ASS deterioration (38.9% vs. 7.4%; *P* = 0.002).

Additionally, the median anti-Jo1 antibody titer was higher in patients who developed ASS deterioration than in those who did not at the time of initial ASS diagnosis (98 vs. 69 U/ml; *P* = 0.033) and last follow-up (135 vs. 47 U/ml; *P* = 0.018) (Table 5).

**Table 5:** **Biochemical parameters associated with antisynthetase syndrome deterioration**^**a**^

**Biochemical parameters**	**ASS remission/improvement**	**ASS deterioration**	***P***
	**(*n*** **= 68)**	**(*n*** **= 18)**	
Overall			
ESR (mm/h)	20 (range: 2 to 108)	32 (range: 8 to 86)	0.09
C-reactive protein (mg/L)	6 (range: 1 to 104)	13 (range: 3 to 212)	0.172
Hemoglobin (g/dl)	13.1 (range: 9.8 to 16)	12.9 (range: 10.2 to 15)	1
Total leukocyte count (giga/L)	6.9 (range: 2.5 to 20.7)	8.3 (range: 3.8 to 25.7)	0.443
Alanine aminotransferase (IU/L)	57 (range: 11 to 368)	45 (range: 15 to 188)	0.763
Aspartate aminotransferase (IU/L)	58 (range: 23 to 787)	70 (range: 12 to 376)	0.537
Creatine kinase (IU/L)	492 (range: 24 to 20,000)	352 (range: 39 to 10,601)	0.861
Anti-Jo1 antibody titer (U/ml)			
At initial diagnosis of ASS	69	98	0.033
At last follow-up of ASS	47	135	0.018

^a^ASS: antisynthetase syndrome; ESR: erythrocyte sedimentation rate. Data are medians (ranges) unless otherwise specified. *P* values were calculated using Fisher’s exact test.

ASS deterioration was not associated with any of the treatments: MTX (*P* = 0.569), AZA (*P* = 0.783), IVIG (*P* = 0.06), MMF (*P* = 0.633) and cyclophosphamide (*P* = 0.106). At the last follow-up, the median daily dose of prednisone was higher in patients with ASS deterioration (17 mg vs. 8 mg; *P* = 0.03). ASS deterioration tended to be more frequent in AZA-treated patients than in the MTX-treated group, although not significantly so (28% vs. 16.7%; *P* = 0.345). The mortality rate was higher in patients who achieved ASS deterioration than among patients who did not (38.9% vs. 7.4%; *P* = 6 × 10^−6^).

## Discussion

Previously, investigators have evaluated PM/DM patient outcomes. They have reported variable rates of PM/DM remission ranging from 25% to 70% [10,14-21]. To date, only a few studies have assessed the outcomes of anti-Jo1 patients with ASS [6,7,22]. In a previous study of 12 anti-Jo1 ASS patients, 41.7% achieved a complete clinical response. The remaining patients exhibited improvement of ASS: Discontinuation of therapy was possible in one patient (8.3%), and long-term therapy was required in the other patients [7]. To the best of our knowledge, our present study represents the largest series of anti-Jo1 patients with ASS. We have found that the prognosis of anti-Jo1 patients with ASS was poorer than that of those without the anti-Jo1 antibody. Only 15.1% of patients in our study achieved ASS remission, whereas clinical status improved in 63.9% and worsened in 20.9%. Our study comprised 86 consecutive patients without prior selection based on clinical presentation, which tends to be representative of anti-Jo1 patients. Our findings are in accord with the data reported by Love *et al*. [6], who found that only 4% of anti-Jo1 patients were relapse-free at the end of their seven-year study period. In our experience, most of the anti-Jo1 patients had a chronic continuous ASS course. Both steroids and cytotoxic drugs could be discontinued in only 4.7% of patients who remained stable after therapy disruption at the last follow-up. Altogether, our data underscore the fact that the presence of anti-Jo1 antibody predicts the prolonged use (longer than three years) of steroids and cytotoxic drugs in ASS patients.

Previous series have included relapse rates of 23% to 60% in PM/DM [10,13,17,23]. In 12 reported anti-Jo1 patients, relapses were frequent in ASS, occurring after steroid therapy withdrawal (91.7%) or during tapering of steroid therapy (41.7%) [7]. Our study interestingly demonstrates that short-term recurrence (occurring during therapy tapering) is frequent in anti-Jo1 patients, being encountered in up to 86% of cases. Among patients who received low-dose steroid therapy (less than 20 mg/day), recurrences occurred in 93.2% of the cases. Long-term recurrence of ASS (after therapy withdrawal) was uncommon (1.2% of patients overall).

PM/DM still has a great impact on patients’ quality of life [10,13,14,19]. In a previously reported series of 87 PM/DM patients, the distribution of HAQ scores revealed that only 17.5% had no disability and 12.5% were severely disabled. The remaining patients (70%) were mildly to moderately disabled [24]. To the best of our knowledge, our present study is the first to evaluate functional disability in anti-Jo1 patients with ASS. In this instance, ASS was strongly associated with decreased functional status in patients. Only 69% of our patients considered to be in remission experienced a return to normal previous activities, whereas the other patients in remission still complained of decreased quality of life (0 < HAQ < 0.75), despite our ability to control active muscle and/or organ disease in these patients. We have further shown that functional outcomes of the nonremitting anti-Jo1 patients with ASS were poor, as shown by the marked reduction of activities (HAQ > 0.75) in 25% of them. Decreased quality of life also occurred due to steroid-related myopathy and/or vertebral compression fracture, which occurred in 36% of our patients.

In two previous series, the 10-year survival rate was 70% to 80% for anti-Jo1 patients [6,25]. In our cohort, we found that the survival rates were 95.3%, 88.9% and 71.4% at one, five and ten years, respectively. The overall mortality rate was 11.6% in our patients, which is higher than that in the general population of the same mean age, in which the five-year mortality rate is about 1% to 2%. Our study further underscores the fact that anti-Jo1 patients with ASS have a 10.5% risk of death due to a cause related to ASS, with death in these patients being due mainly to cancer and pulmonary complications.

High-dose oral prednisone is the mainstay of therapy for ASS patients [23]. In patients who fail to respond to prednisone alone (that is, patients with deterioration of ASS clinical manifestations and increased CK levels after at least four to eight weeks), the first-line therapy comprises MTX and AZA [23]. Previous authors have observed that ASS patients exhibited a better response to MTX than to AZA [22,26]. Our findings are in accord with these data, as we observed that MTX treatment tended to result more frequently in ASS improvement than AZA therapy in anti-Jo1 patients with ASS. Moreover, MTX has the advantage of being faster-acting than AZA, allowing improvement of muscle strength and CK levels six to eight weeks after therapy initiation [23]. In our experience, the proportion of ILD patients treated with MTX and AZA did not differ. Nevertheless, MTX therapy necessitates close monitoring in patients with ILD. Because of the risk of MTX-associated lung injury, such therapy is not recommended in the subgroup of patients with severe ILD (on the basis of PFTs and HRCT scans). IVIG therapy should be considered in the following groups of patients. One is patients who fail to respond to a combination of prednisone, MTX or AZA treatment. IVIGs have been shown to be a useful therapy in refractory PM/DM, with reported improvement rates ranging from 67% to 92% [23]. In this instance, IVIGs resulted in improvement of ASS in 70% of patients. We have also observed, interestingly, that patients who exhibited ASS resolution or improvement, compared with those who did not, more often tended to be those treated with IVIGs, although not significantly so (*P* = 0.06). A second patient group for whom IVIG therapy should be considered comprises those for whom cytotoxic drugs are contraindicated. Another group comprises those patients who have esophageal involvement [23]. In a retrospective series, 73 patients with steroid-refractory esophageal involvement related to PM/DM received IVIG therapy. Sixty of the IVIG-treated patients in that series had rapid resolution of clinical manifestations related to esophageal dysfunction (82.2%) [27]. In our current study, we found that IVIG therapy led to improvement of esophageal involvement in 90% of patients. We further suggest that, in patients with severe esophageal manifestations, combined high-dose steroids and IVIGs might be proposed as the first-line therapy. To date, in patients who fail to respond to a combination of prednisone, MTX/AZA and IVIG, the first-line treatment option has included rituximab. On the basis of data obtained from case reports and open series, rituximab has been shown to be effective in patients with PM/DM refractory to other therapies [23]. In a recent randomized series, 200 patients (76 with PM, 76 with DM and 48 with juvenile DM) received rituximab and corticosteroids and immunosuppressive therapy were allowed at study entry [28]. The authors of that study found that 83% of the rituximab-treated patients had improvement of clinical manifestations at the 44-week follow-up [28]. In our experience, one patient with refractory ASS and ILD was successfully treated with rituximab.

Current therapy for ILD in ASS is based on the use of steroids [3,12]. Previous studies have found a better response to steroid therapy in anti-Jo1 patients with NSIP and COP compared with those with UIP [3,12]. In this instance, ASS patients with UIP had a worse prognosis than patients with NSIP and/or COP. Cyclophosphamide may improve the clinical outcomes in these patients [3,12,23]. In a retrospective study, cyclophosphamide (three to six cycles intravenously) was used in 25 PM/DM patients with ILD, which resulted in resolution (24%) or improvement (40%) of pulmonary status [3]. The present series also suggests that pulse cyclophosphamide is useful in ASS patients with ILD, resulting in resolution or improvement of pulmonary status in 89.4% of cases. We further suggest that patients with factors predictive of ILD deterioration (that is, UIP pattern on HRCT scans) may require combined therapy with prednisone and cyclophosphamide. Additionally, rituximab has been suggested to be effective in PM/DM patients with refractory ILD [2,3]. In our experience, one patient with refractory myositis and ILD was successfully treated with rituximab as follows: 1 g at days 0 and 14 and 1 g every 6 months for 18 months. To date, there is anecdotal evidence that tacrolimus is effective in ASS patients with refractory ASS and ILD [23].

From a practical point of view, knowledge of prognostic factors regarding the ASS course is crucial. The most important predictor of mortality is age, with older PM/DM patients having a poorer prognosis [10,15-18,24,26,29]. In this instance, older age (60.5 vs. 53 years) was correlated with ASS deterioration data in anti-Jo1 patients.

Esophageal involvement has been associated with higher mortality in PM/DM patients [10,15,17,27]. Respiratory muscle involvement also has been described to be a factor predictive of death in PM/DM patients [12,13,15,29,30]. In this instance, predictive factors of ASS deterioration in anti-Jo1 patients were muscle weakness, esophageal impairment, ventilatory insufficiency related to striated muscle weakness and aspiration pneumonia. Our findings suggest that this group of patients may require aggressive therapy with short-term efficacy. In addition, our findings show that calcinosis cutis may be a factor predictive of poor outcome, resulting in disabling functional and esthetic complications.

Another main observation can be drawn from our study. Previous authors have found an increased risk of cancer among DM patients (odds ratio (OR): 6.2, 95% confidence interval (CI): 3.9 to 10.0) and PM patients (OR: 2.0, 95% CI: 1.4 to 2.7). Although the risk of cancer was highest around the time of PM/DM diagnosis, the increased risk persisted beyond five years (OR: 1.6, 95% CI: 1.0 to 2.6) [31]. Cancer-associated myositis in the presence of ASS has also been reported [32-34]. In a series of 95 ASS patients, patients with the anti-Jo1 antibody more commonly had cancer than those with the anti-threonyl-tRNA synthetase (anti-PL7)/PL12 antibody [3]. In this instance, we found that up to 13.9% of anti-Jo1 patients with ASS developed cancer either concurrently or within three years before or after PM/DM onset. Our findings interestingly underscore the fact that the prevalence of cancer was higher in our 86 anti-Jo1 patients with ASS than in age- and sex-matched individuals in the general population (OR: 6.75, 95% CI: 1.4319 to 64.081; *P* = 0.009). We therefore suggest that anti-Jo1 patients with ASS (especially those older than 50 years of age) require evaluation for cancer. Interestingly, 33.3% and 50% of our patients with cancer developed digestive (mainly colon) and gynecologic (ovarian, uterine and breast) cancers, respectively. Furthermore, cancer is considered to be a factor contributing to a poor prognosis in PM/DM patients [10,14,15,21,35,36]. In a previous study of 197 PM/DM patients, cancer was positively associated with mortality (hazard ratio: 2.30, 95% CI: 1.26 to 4.22; *P* = 0.003) [34]. Our present study shows that cancer is highly associated with ASS deterioration in anti-Jo1 patients (*P* = 0.002), and five patients died of cancer.

The last relevant finding in our study is that the level of anti-Jo1 antibody was higher in the group of patients who exhibited ASS deterioration at initial evaluation and last follow-up. We suggest that high anti-Jo1 levels may be a factor predictive of ASS deterioration. However, further investigations are required to confirm our findings.

## Conclusion

Our large study shows high morbidity related to ASS in anti-Jo1 patients. Only 15.1% of patients achieved remission of ASS, and both steroids and cytotoxic drugs could be discontinued in 4.7% of cases. Furthermore, we suggest that patients with factors predictive of ASS deterioration may require more aggressive therapy. Our findings also suggest that in anti-Jo1 patients with severe esophageal manifestations, combined high-dose steroids and IVIGs might be proposed as the first-line therapy. Cancer occurred in up to 14% of our patients, so we think a search for cancer should be performed in anti-Jo1 patients with ASS.

## Abbreviations

ASS: Antisynthetase syndrome; AZA: Azathioprine; CK: Creatine kinase; DLCO: Diffusing capacity of carbon monoxide; HAQ: Health assessment questionnaire; HRCT: High-resolution computed tomography; ILD: Interstitial lung disease; IVIG: Intravenous immunoglobulin; MF: Mycophenolate mofetil; MMT: Manual muscle strength test; MTX: Methotrexate; PFT: Pulmonary function test; PM/DM: Polymyositis/dermatomyositis.

## Competing interests

This work was not supported by any funding or sponsors. Moreover, the authors do not have any financial or personal relationships with other people or organizations that could create a potential conflict of interest or the appearance of a conflict of interest with regard to the work.

## Authors’ contributions

IM and SD were responsible for the study’s conception and design. IM, PYH, PC, EH, ED, OV, JFM, FJ and SD contributed to data acquisition. IM and JFM performed data analysis. IM, JFM and SD contributed to data interpretation. IM and SD drafted the manuscript. PYH, PC, EH, ED, OV, JFM and FJ revised the manuscript critically for important intellectual content. All authors gave their final approval of the version of the manuscript to be published.
